# Hillock assisted p-type enhancement in N-polar GaN:Mg films grown by MOCVD

**DOI:** 10.1038/s41598-020-58275-1

**Published:** 2020-01-29

**Authors:** Emma Rocco, Olivia Licata, Isra Mahaboob, Kasey Hogan, Sean Tozier, Vincent Meyers, Benjamin McEwen, Steven Novak, Baishakhi Mazumder, Michael Reshchikov, L. Douglas Bell, F. Shahedipour-Sandvik

**Affiliations:** 1 College of Nanoscale Science and Engineering, SUNY Polytechnic Institute, Albany, NY USA; 2 Department of Materials Design and Innovation, University at Buffalo, Buffalo, NY USA; 3 Department of Physics, Virginia Commonwealth University, Richmond, VA USA; 4 Jet Propulsion Laboratory, California Institute of Technology, Pasadena, CA USA

**Keywords:** Electronic devices, Electronic properties and materials

## Abstract

We report on the enhanced incorporation efficiency of magnesium dopants into facets of hexagonal hillock structures in N-polar GaN, studied by comparative analysis of GaN:Mg films grown by MOCVD on high and low hillock density GaN template layers. Total magnesium concentration in planar regions surrounding a hillock structure is comparable to that within hillock sidewall facets measured at 1.3 × 10^19^ cm^−3^ by atom probe tomography, and clustering of Mg atoms is seen in all regions of the film. Within individual hillock structures a decreased Mg cluster density is observed within hillock structures as opposed to the planar regions surrounding a hillock. Additionally, the Mg cluster radius is decreased within the hillock sidewall. The favorable incorporation of Mg is attributed to Mg dopants incorporating substitutionally for Ga during growth of semi-polar facets of the hillock structures. Enhanced p-type conductivity of GaN:Mg films grown on high hillock density template layers is verified by optical and electrical measurement.

## Introduction

The gallium nitride (GaN)-based material system and its ternary and quaternary alloys with aluminum (Al) and indium (In) are widely employed in light emitting diodes (LEDs), photodetectors^1–3^, and the next generation of power devices^4–7^. Due to the lack of inversion symmetry within the III-Nitride wurtzite crystal structure the material exhibits a spontaneous polarization charge along the c-direction. The GaN crystal can be grown in the Ga-polar (0001), N-polar (000$\overline{1}$), semi-polar or non-polar (1$\overline{1}$00), (11$\overline{2}$0) orientation. While the Ga-polar orientation has been most extensively studied, the N-polarity has been shown to enhance hole injection^8^ and decrease efficiency droop^9^ in LEDs, and increase quantum efficiency^3^.

Growth of N-polar GaN differs from that of Ga-polar due to differences in the adatom diffusion barrier^10^. The formation of hexagonal^11^ and triangular^12^ hillocks are common in N-polar GaN growth on sapphire and SiC substrates, respectively. The formation of hillocks during N-polar growth on sapphire substrates has been attributed to oxygen rich domains produced by electromechanical polishing forming inversion domains^13^ and the lower adatom mobility of Ga due to lack of a Ga adlayer during growth on (000$\overline{1}$) surfaces^10^. Hillock structures consist of terraced semi-polar sidewalls. The size of hexagonal hillocks is greatly dependent on growth conditions and can be on the scale of microns in both width and height^14^.

Suppression of hillocks during growth on sapphire substrate is possible through utilization of off-cut substrates, addition of an AlN nucleation layer and use of In surfactant in the GaN growth layer^11^. The AlN nucleation layer conditions are critical in order to establish N-polar growth and achieve high crystal quality. AlN nucleation layers grown at temperatures above 850 °C have been shown to establish an N-polar crystal orientation^15^. With increasing AlN growth temperature up to 1150 °C the material quality increases as measured by x-ray diffraction, and RMS roughness decreases for the overgrown N-polar GaN layer^15^. Additionally, Indium surfactant has been shown to greatly reduce hillock density with increasing In flow rate^16^. Density functional total energy calculations have shown that In modifies the surface energy and adatom diffusion barriers of Ga and N^17^, while minimal In is incorporated into the film at the high growth temperatures used for GaN growth.

Mg is the most common p-type dopant in the III-nitride material system due in part to an established activation process^18^. However, challenges remain to obtaining high conductivity p-type films utilizing Mg as the dopant due to a high ionization energy, the formation of compensating nitrogen vacancies^19^, and segregation of Mg into clusters that may be electrically inactive^20^. Additionally, the N-polarity suffers from a high concentration of unintentionally incorporated oxygen (compared to the Ga-polarity), which compensates free holes^21^. Progress has been made in partially overcoming these challenges through the use of delta doping in both the Ga- and N-polarity^22–24^ to enhance p-conductivity. Both calcium^25^ and zinc^26–30^ have been explored as alternative acceptors in GaN however, the ionization energies in both dopant species have been shown to be similar or higher compared to magnesium. Beryllium (Be) is expected to be a shallow acceptor dopant in GaN based on theoretical calculation studies and some experimental demonstration through limited growth studies^31^. However, the reproducibility of these results and achieving reasonable p-type conductivity have been difficult in large part to self-compensation by Be^32^. Theoretical studies predict a low formation energy for interstitial Be which act as donors compensating the Be acceptors^32^.

Dopant clustering is common in many material systems when the concentration of dopants exceeds the solid solubility. Atomic size, valency and electronegativity are among the factors determining the solid solubility of a dopant in a material^33^. Mg has been shown in Ga-polar GaN grown by MOCVD to form clusters at high dopant concentrations above 1 × 10^19^ cm^−3^, which have been shown to impact the optical properties of the film^20^. In addition to Mg, Manganese (Mn) doping in GaN grown by MBE has been studied for ferromagnetic properties in spintronic applications and has been shown to form clusters^34,35^. Conversely, donor dopants such as Si and Ge have not been shown to precipitate into clusters in GaN^36^.

High hole concentrations have been realized in semi-polar GaN:Mg films grown by metal organic chemical vapor deposition (MOCVD), with free hole concentration of 2.4 × 10^18^ cm^−3^ achieved in (10$$\overline{1}$$$$\overline{1}$$) films^37^. Such experimental results have been confirmed by first-principles pseudopotential calculations which confirm an increased incorporation of Mg_Ga_ into electrically active sites in semi-polar films with a high density of step edges^38^.

We report here for the first-time differential incorporation of Mg dopants within hillock structures compared to planar N-polar films through a contrast in Mg cluster density and size. We demonstrate the dependence of Mg incorporation site on the plane of incorporation. Hillock structures are shown to preferentially incorporate Mg with lower cluster density, which is attributed to the presence of the semi-polar sidewalls. Additionally, through correlation between the electrical and optical properties and density of Mg clustering we show that p-type conductivity is enhanced for GaN:Mg films grown on template layers with a high hillock density. The favorable incorporation of Mg within hillock structures and resulting enhanced p-type conductivity can be utilized to improve device efficiency for a wide variety of applications.

## Experimental Methods

**Figure 1:**
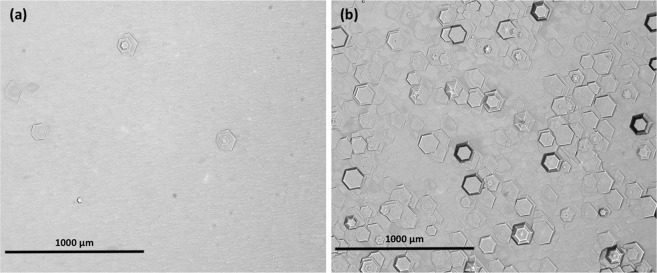
Optical micrographs of (**a**) low hillock and (**b**) high hillock density N-polar GaN template layers. Scale bar is equivalent.

GaN structures were grown by MOCVD on nominally on-axis sapphire substrates with a miscut of 0.2° toward m-plane (1$\overline{1}$00). Unintentionally doped N-polar GaN template layers were grown to a nominal thickness of 450 nm using a two-step AlN buffer and high temperature GaN film using conditions reported previously^11^. Low hillock density N-polar GaN templates were achieved through the use of an optimized aluminum nitride (AlN) buffer and indium (In) surfactant^11^. High hillock density films resulted from growth without In surfactant and increased growth time of the high temperature GaN layer by 25%. Low and high hillock density films resulted in an average 10 hillock/cm^2^ and 500 hillocks/cm^2^, respectively, and shown in Fig. 1. Hereafter, high hillock density template is referred to as HHT, and low hillock density template as LHT.

In order to study the incorporation of Mg dopants and its potential dependency on hillocks, GaN:Mg layers were overgrown on the high and low hillock density template layers under identical growth conditions. The overgrowth consisted of 450 nm GaN:Mg followed by 10 nm uGaN cap layers. Trimethyl gallium (TMGa) and ammonia (NH_3_) precursors at flow rates of 65 μmol/min and 845 μmol/min were used respectively. Bis-cyclopentadienyl-magnesium (Cp_2_Mg) was utilized as the p-type dopant at 370 nmol/min flow rate. Following completion of growth, samples were annealed at 775 °C for 15 minutes in nitrogen ambient to activate Mg.

Mg dopant concentrations of the films were studied by dynamic secondary ion mass spectroscopy (SIMS) utilizing a Phi 6650 quadrupole mass spectrometer employing a 5 keV Cs^+^ ion bombardment at 60 degrees incidence from normal. Time-of-Flight (TOF) SIMS measurement was acquired using an IonToF ToF-SIMS V-300 using bismuth positive ion detection analysis beam of 5 μm diameter at 45 degrees incidence from normal. Mass resolution of TOF-SIMS was sufficient to eliminate any peak overlaps. Nanoscale chemistry and Mg distribution were measured by atom probe tomography (APT) using a CAMECA LEAP-5000 XR, equipped with reflectron lens and ultraviolet (λ = 355 nm) laser pulsing capabilities. APT specimens were prepared from site-specific regions including planar (surrounding hillocks) and hillock sidewall structures. Standard lift out procedures for APT specimens were performed in an FEI DualBeam 875 focused ion beam (FIB) to create needles with a tip radius smaller than 100 nm. APT analyses were carried out at a base temperature of 30 K, with a pulse energy of 4–5 pJ and detection rate of 0.005–0.008 ions per laser pulse.

**Figure 2:**
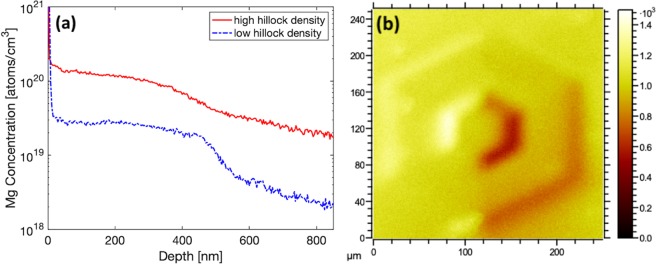
(**a**) Mg SIMS scan of GaN:Mg layers grown on low (dashed) and high (solid) hillock density template layers, (**b**) TOF-SIMS image of all ions collected from a single hillock structure shown on a thermal scale without depth profile sputter.

Photoluminescence spectroscopy was completed to characterize the optical properties of the material using a HeCd laser with 50 mW of power, Triax 320 monochrometer, cooled photomultiplier tube, and optical closed-cycle cryostat. Ionized acceptor concentration for each of the samples was determined by MDC Hg probe capacitance-voltage (C-V) at 100 kHz frequency with a dot contact diameter of 760 μm.

## Results and Discussion

The concentration of Mg as a function of depth was measured by SIMS and shown in Fig. 2a. In the film grown on HHT layer, the average Mg concentration in the doped layer is measured to be 1.2 × 10^20^ cm^−3^. The film grown on LHT achieved an average Mg concentration of 2.5 × 10^19^ cm^−3^. These results indicate that on average nearly an order of magnitude increased incorporation of Mg in the GaN:Mg layer grown on HHT. However, the large size of the hillock structures present challenges for site specificity in SIMS measurement.

**Figure 3:**
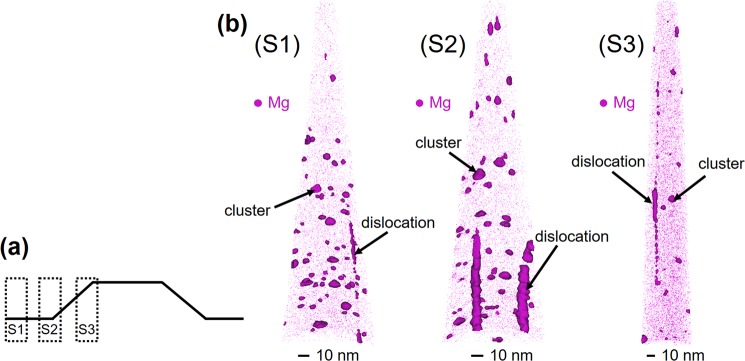
APT reconstructions of tips from (S1) planar region surrounding hillock structure, (S2) beginning of hillock sidewall, and (S3) top of hillock sidewall. 3D atom maps include dark volumes encapsulating regions of segregation (clusters). Continuous dislocations were excluded for concentration and cluster number density calculations.

The data collection area of the sample in dynamic SIMS measurement is 100 μm^2^ and thus the concentration measured is an average from this area. Additionally, for samples measured here, multiple hillocks may be present within the measurement area. The size of the hillock structures and the angle of incidence of the sputtering beam pose unique challenges to interpreting SIMS depth profile results. The hexagonal hillock structures have dimensions on the order of 100 μm in diameter and 100 nm in height. During the sputtering process the hillocks create a shadowing effect wherein the peak of a hillock structure may prevent the sputtering and ionization of atoms in the valley between hillock structures. This can be visualized in spatially resolved TOF-SIMS in Fig. 2b. A single hillock structure is measured for the combined intensity of all ions. On the right side of the hillock structure a lower intensity of ions is measured as shown by the dark color of the thermal scale, compared to the left side of the hillock.

The shadowing mechanism that is depicted by the TOF-SIMS image (Fig. 2b) contributes to the shape of the dynamic SIMS depth profile. Beyond the intentionally Mg doped layer, both samples are expected to have identical background Mg concentration. However, a higher concentration of Mg within the unintentionally doped layer is seen in the high hillock density sample. This can be explained by the shadowing effect of the hillocks during sputtering. As a hillock peak is sputtered decreasing in height, the shadowed area between hillock structures will be revealed to the ionizing beam. This leads to extended measurement of the intentionally doped Mg layer throughout the depth of the expected unintentionally doped layer.

In compliment to the SIMS measurements on the micron scale, APT has been employed for nanoscale characterization of Mg concentration and distribution profile. APT provides the resolution necessary to compare the Mg distribution profile between the planar region surrounding a hillock structure and the hillock side wall. The cross-sectional schematic in Fig. 3a shows the three sample regions spanning a single hillock structure from the HHT sample. The Mg distribution 3D atom maps, obtained from APT analysis, are shown in Fig. 3b. Clustering of Mg atoms is observed in all three regions and indicated in Fig. 3b with isoconcentration surfaces generated in Integrated Visualization and Analysis Software (IVAS). The isoconcentration surfaces shown in (S1) and (S3) have threshold concentration values (isovalues) of 1.0% Mg and (S2) has an isovalue of 1.2% Mg. Hillocks from both HHT and LHT were sampled for APT and showed similar results of bulk Mg concentration and cluster density. The clustering phenomenon has been attributed to the concentration of Mg reaching the solid solubility limit in GaN^39^, and segregation of solutes at dislocations^40^. The 3D atom maps suggest a variation in the distribution and size of Mg clusters.

**Figure 4:**
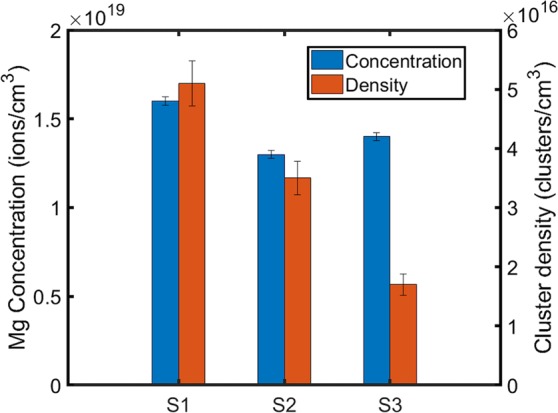
Bar plot comparing Mg cluster number density and bulk concentration of Mg for all three sample tips. The standard error for each measurement is shown with error bars.

The bulk Mg concentration was measured by APT and found within a range of 1.3–1.6 × 10^19^ atoms/cm^3^ for the three regions, such that the total Mg concentration is nominally equivalent in planar regions and hillock regions. The number density of Mg clusters was estimated from the number of fully-contained plus half the partially-contained clusters obtained through isosurface characterization in IVAS and following established methods in literature^41–43^. The number density of Mg clusters from the planar region (S1) towards the center of the hillock sidewall (S3) demonstrated a decreasing trend outside of error, as shown in Fig. 4.

**Figure 5:**
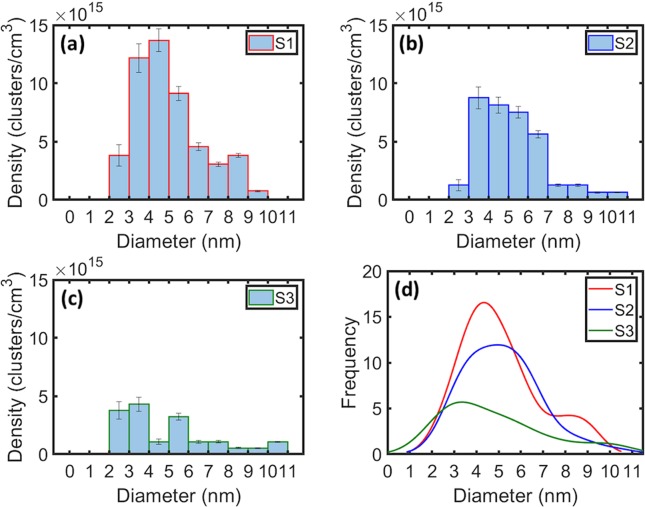
Comparison of the cluster number densities with cluster size in samples **(a)** S1 **(b)** S2 and **(c)** S3. **(d)** Kernel distribution fits from comparison of cluster size and frequency.

The volumes of Mg clusters, estimated from isosurfaces, were converted to their effective diameters, assuming spherical geometry. Clusters within the dislocations could not be accurately measured and were thus excluded from the calculations of number density and size. Outside of the hillock structure in the planar region (S1) the average radius of the clusters is 5.1 nm. The average cluster radius decreases to 4.8 nm within the hillock sidewall (S3). The distribution of cluster sizes from the three sample regions varies, as shown in Fig. 5a–c; however, they demonstrate similar mean values when fit with a normal distribution. In order to capture the skew in cluster size distributions, a kernel distribution was obtained. Figure 5d directly compares the respective kernel distribution fits of cluster sizes from the three sample regions. Their individual distributions vary in spread and tend to broaden from the planar region (S1) towards the hillock sidewall (S3), indicating fewer and smaller-sized clusters within the hillock structure.

Given the results of APT analysis, while the total amount of Mg incorporated in planar and hillock regions is equal, Mg is incorporated differently into the film in the planar and hillock regions. Favorable Mg incorporation is observed in the hillock sidewall with homogenous Mg distribution. The reduction in cluster density and size within the hillock sidewall results in fewer dopants in electrically inactive clusters. We attribute the favorable incorporation of Mg within hillock structures to the presence of semi-polar facets and nano-facets on the hillock sidewalls. Presence of nano-faceting on the sidewalls has been confirmed by TEM analysis (not shown here). First principle pseudopotential calculations have shown that Mg will be preferentially incorporated substitutionally for Ga atoms at the step edges of semi-polar (10$$\overline{1}$$$$\overline{1}$$) GaN^38^. Further, the surface energy of the semi-polar plane which contains a single substitutional Mg atom was calculated to be lower compared to the surface incorporating two Mg atoms at the site^38^. The hillock structures reported here provide preferential sites and favorable kinetics for incorporation of Mg in substitutional sites. According to the theoretical work of Akiyama *et al*. the energetically favorable incorporation of Mg into the semi-polar planes promotes the incorporation of a single Mg atom at a Ga site as opposed to multiple Mg atoms, or Mg incorporating in interstitial sites. The increased uniform distribution of Mg throughout the lattice of the hillock structures increases the probability of Mg substitutionally occupying an electrically active Ga site and the likelihood of higher free hole concentration in high hillock density samples.

**Figure 6:**
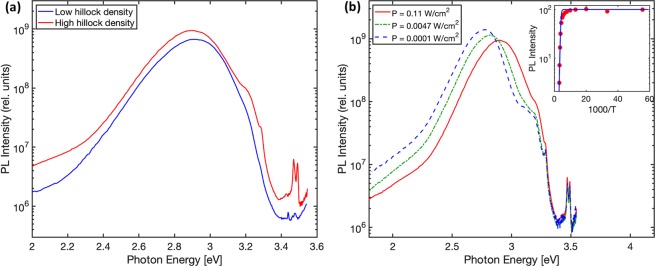
(**a**)Photoluminescence spectra of GaN:Mg films grown on low (blue) and high (red) hillock density template layers utilizing 0.11 W/cm^2^ excitation power density at 18 K, and (**b**) power intensity dependent photoluminescence spectra of GaN:Mg film grown on high hillock density template at 18 K with inset showing temperature dependence behavior of “blue band” intensity (red dots) with fit to Eq. (1) (blue solid line).

Photoluminescence spectroscopy was performed on GaN:Mg films deposited on both HHT and LHT samples. Low temperature photoluminescence spectra taken at 18 K and 0.11 W-cm^−2^ excitation power density are shown in Fig. 6a. In both spectra, a broad so-called “blue band” is present at 2.8–2.9 eV consistent with the PL signature for p-type GaN^44^ grown by MOCVD. The blue band (BB) in highly Mg doped samples arises from a transition from an unknown deep donor state to a shallow Mg acceptor state. In Fig. 6b the excitation power dependent spectra for the film grown on high hillock density template is shown. The blue shift of the BB with excitation intensity is also consistent with this transition and is due to saturation of distant donor-acceptor pairs and increasing emission from close pairs with stronger Coulomb interaction^44^.

The BB is quenched above 200 K which has been attributed to thermal emission of electrons from the deep donor state to the conduction band^45^. The temperature dependence of the BB photoluminescence intensity for the HHT sample is shown in the inset of Fig. 6b. The dependence is fitted with the Arrhenius function,

1 $$I(T)=\frac{{I}_{0}}{1+{\rm{Aexp}}(-{E}_{A}/{k}_{B}T)}$$

where I_0_ is the intensity at low temperature, A is a temperature independent constant and E_A_ is the activation energy. Through this equation an activation energy of 0.3 eV is calculated which is consistent with values reported previously^44–46^.

Other peaks present in the PL spectra are donor bound exciton (DBE) at 3.490 eV, acceptor bound excitons (ABE) at 3.470 eV, UV band at ~3.1 eV and UVL band at 3.285 eV. The UVL band is due to transitions from the conduction band to the shallow Mg acceptor state. The UV band may be the same transition as the UVL band from regions with strong electric fields, such as the near surface region. The strong ABE and BB are indicative of regions of p-type material, while the UVL and DBE may arise from high-resistivity regions or n-type regions.

**Figure 7:**
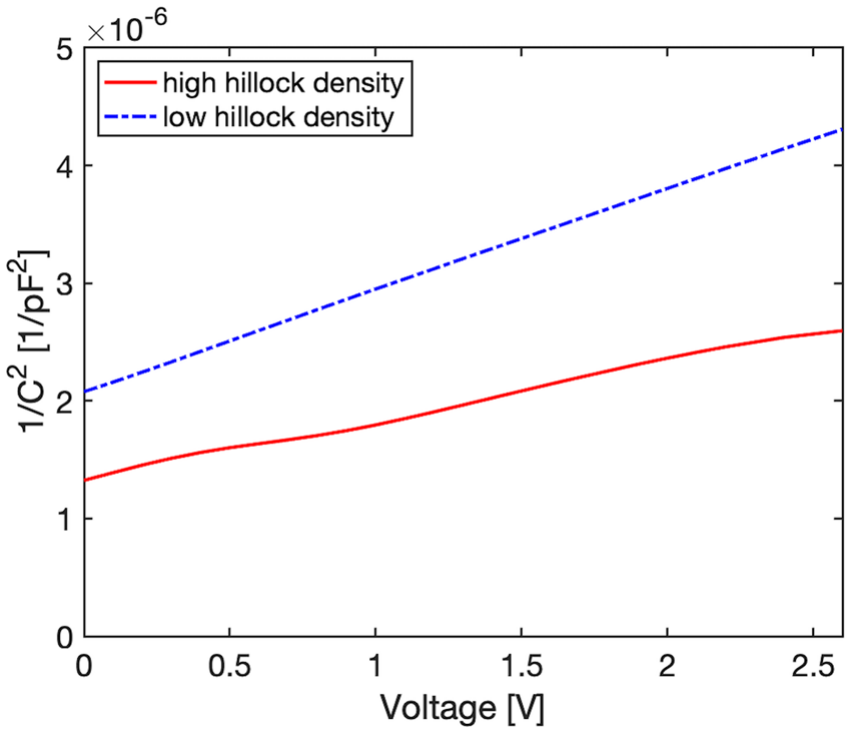
Plots of 1/C^2^ vs. V, measured by Hg probe C-V, of GaN:Mg layers grown on high and low hillock density GaN template layers.

To assess the impact of Mg incorporation and distribution differences on electrical properties of the material, mercury probe C-V measurements were performed. Measurement of 1/C^2^ vs. V are shown in Fig. 7 for GaN:Mg films grown on HHT and LHT samples. From the slope of 1/C^2^ vs. V, N_A_-N_D_ is extracted with the GaN:Mg film grown on HHT measured at 1.61 × 10^18^ cm^−3^. Comparatively, the GaN:Mg film grown on LHT layer is 8.26 × 10^17^ cm^−3^. Assuming a Mg concentration of 1.3 × 10^19^ cm^−3^ given by APT, a 2x increase in hole concentration is calculated for the GaN:Mg film grown on HHT. Density functional theory calculations suggest a lower stress for Mg atoms incorporated at step edges of the semi-polar (10$$\overline{1}$$$$\overline{1}$$) GaN^38^ which may lead to reducing the formation of V_N_, that act as compensating donors^19^. The increased hole concentration of GaN:Mg films grown on HHT is attributed to the increased substitutional Mg incorporation at electrically active Ga sites, decreased Mg incorporation into clusters, and a decreased formation of compensating defects^20,37,38^.

## Conclusions

The incorporation of Mg into planar N-polar and semi-polar hillock sidewall facets has been studied experimentally. Measurement of Mg concentration and distribution by atom probe tomography shows similar total bulk Mg incorporation in planar regions surrounding hillocks and within hillocks with semi-polar facets. The Mg cluster density and radius decreases as a function of increasing distance from the planar region toward the hillock sidewalls. Higher uniformity in distribution of Mg within the matrix is attributed to the favorable kinetics of Mg_Ga_ incorporation in the semi-polar planes of the hillock sidewalls. The increased probability of Mg incorporation into electrically active Ga sites is proposed as the mechanism for the increased p-type conductivity of GaN:Mg films grown on high hillock density template layers. Based on the results of APT and CV measurements, an enhancement in hole concentration is expected for GaN:Mg films grown on high hillock density template layers. Improved Mg incorporation efficiency in N-polar GaN films reported here offers a novel way to enhance performance in devices such as UV photodetectors and emitters where p-conductivity is particularly an issue.

## Data Availability

The data that support the finding of this study are available from the corresponding author upon reasonable request.
